# Evaluating the new product Norroa™ against *Varroa destructor* in managed honey bee (*Apis mellifera*) colonies

**DOI:** 10.3389/finsc.2026.1751606

**Published:** 2026-03-11

**Authors:** Devan Rawn, Cody Prouty, Asmita Gautam, Matthew Jamison, Win Talton, Katie Youngs, Ken Narva, Brian Manley, Cameron Jack

**Affiliations:** 1 Entomology and Nematology Department, University of Florida, Gainesville, FL, United States; 2 GreenLight Biosciences, Research Triangle Park, Durham, NC, United States

**Keywords:** *Apis mellifera*, honey bee, Norroa, RNAi, vadescana

## Abstract

**Introduction:**

Globally, beekeepers must manage the invasive mite pest *Varroa destructor*. The mite’s populations can grow quickly, overwhelming honey bee colonies through direct parasitism and the transmission of honey bee viruses. RNA interference is promising next-generation tool and has been demonstrated to control invertebrate pest populations. The novel product Norroa™ is the first of its kind marketed to beekeepers for the control of *V. destructor*. Field-level studies are critical to understanding the efficacy of the product and how to fit it into integrated pest management plans.

**Methods:**

Field studies were conducted to test the effectiveness of Norroa™ during a nectar-flow season and a nectar-dearth season in Florida. In each field trial, 36 colonies were established for testing. Mite infestation rates were measured throughout the trials and mites were assessed for gene knockdown by determining RNA concentrations from the target gene. A final trial was conducted uncapping honey bee pupae and examining mite reproduction.

**Results:**

During the nectar dearth, mite numbers started higher (4.56 mites/100 bees) and increased more in control colonies compared with the treated colonies, but differences were not significant. During the nectar-flow, Norroa™ maintained *V. destructor* populations at or below the initial infestation rate (2.31 mites/100 bees) for twelve weeks, compared with the control group that grew significantly higher than treated colonies. Mites from colonies that had been treated with Norroa™ were significantly less likely to lay an egg, or have any offspring emerge from the eggs laid.

**Discussion:**

Research related to biological methods of control are currently under-studied relative to chemical treatments. These trials demonstrate the promise of a new tool for beekeepers to control *V. destructor* as part of a sustainable IPM approach.

## 1 Introduction

*Varroa destructor* is a parasitic mite and is considered by many beekeepers worldwide to be the most destructive honey bee (*Apis mellifera*) pest (1). The primary damage caused by the mite is linked to its ability to vector and transmit honey bee viruses (2) sometimes leading to massive economic losses (3). This mite’s populations can grow rapidly, especially in the summer and fall seasons (4). *Varroa destructor* now has a worldwide distribution and can be found nearly everywhere honey bees are managed (5). The reproductive phase of *V. destructor’s* life cycle is initiated when female mites immerse themselves in brood food beneath 5^th^ instar honey bee larvae and remain undetected until the larval cell is sealed (6).

Beekeepers can combat *V. destructor* optimally by using integrated pest management (IPM) that involves multiple active ingredients and control methods including cultural, mechanical and biological methods without relying on a single product (7). There is a need in the beekeeping industry to diversify reliance on synthetic acaricides because of resistance to amitraz (8, 9), *tau*-fluvalinate (10, 11), and coumaphos (12, 13). Options exist for acaricides made from organic acids and other botanically derived volatile compounds, but their use and efficacy can be limited by climactic conditions and colony conditions (14).

RNA interference (RNAi) is a promising next-generation technology that has shown potential in managing invertebrate pest populations (15–22). This approach leverages the specificity of double-stranded RNA (dsRNA) to selectively silence genes critical for the survival and reproductive processes of target pest species. Laboratory testing on small populations of bees and mites has shown the safety of RNAi methods to bees and the ability to reduce infestations of *V. destructor* (23, 24). Earlier research in using dsRNA to silence target genes in *V. destructor* have used techniques of direct injection of the dsRNA into the mite (25) or through ingestion (23). More recently, researchers have been able to expose the mites to the dsRNA through immersion in solution containing the dsRNA (26). This route of exposure may be realistic within a hive environment, as *V. destructor* can be immersed in brood food before it begins reproducing (24). Through surveys, researchers have seen support amongst beekeepers for RNAi technology to control *V. destructor* (27).

Several genes that are related to *V. destructor* survival or reproduction have been identified as potential candidates for RNAi targeting (28). Field trials have demonstrated that RNAi can be used to prevent honey bees from succumbing to infections of viruses (29). Other trials using RNAi to limit *V. destructor* infestation have shown potential when targeting *V. destructor* acetyl-CoA carboxylase gene for silencing (26). Recently, a dsRNA that targets a calmodulin gene, calmodulin-like, in *V. destructor*, which is responsible for encoding the calmodulin protein, has been developed by GreenLight Biosciences, Inc. This *V. destructor*-specific dsRNA is referred to as ‘vadescana’ (CAS# 2643947-26-4) and has demonstrated the ability to safely control the mite by down-regulating a calmodulin gene and reducing the calmodulin protein levels in mites. This dsRNA is 402 bp long and it targets the *V. destructor* calmodulin homolog gene, “calmodulin-like” (GenBank accession XM O22799184.1). Off-target effects of this specific dsRNA sequence have been investigated (30) and it has been demonstrated to be highly specific to *V.* destructor. Calmodulin is involved in a number of processes in arthropods including reproduction. Studies where dsRNA targeting the calmodulin gene was microinjected into nymphs observed suppressed ovary development leading to reduced fecundity and lethal molting deformities in *Nilaparvata lugens* (Hemiptera: Delphacidae) (31). Laboratory trials of vadescana have demonstrated that this dsRNA affects *V. destructor* fecundity, presenting new options for control (24). When using the BlastN tool through NCBI (blast.ncbi.nlm.nih.gov) and searching for significant alignment matches to our target gene, no *Apis mellifera* sequences show a similarity above 80% alignment. The closest off-target organism match is *N. lugens* calmodulin at an 85.71% alignment.

Here, we tested the efficacy of vadescana in the field through a series of tests using the product Norroa™, created by GreenLight Biosciences Inc. Our primary goals with the research presented herein was to determine the impact of colony size and nectar flow on treatment efficacy of Norroa™. Biological approaches to pest management may be less reliably effective against *Varroa destructor* when compared to synthetic acaricides (32) and careful fine-tuning of dose and delivery may be required. Secondarily, we wanted to trace the product in the hive to confirm optimal *V. destructor* exposure, and observe direct effects of Norroa™ on reproduction. The results from these experiments allow us to understand the most effective ways to apply the Norroa™ product for maximum efficacy against *V. destructor*.

## 2 Methods

### 2.1 Experiment 1

#### 2.1.1 Field efficacy of Norroa™

Two full field trials were conducted throughout this project. All field trials took place at the UF/IFAS Plant Science and Education Unit (29°24’33” N 82°08’53” W) in Citra (Marion County), Florida. To test the efficacy of Norroa™ during a nectar dearth, we conducted a trial in September of 2023, when little or no nectar is available to honey bee colonies at this location (nectar-dearth trial). The second trial was conducted in May 2024 during a time of abundant nectar availability in the area (nectar-flow trial). During the winter and spring between field trials, colonies from the trial location were removed and new colonies were brought in. In both field trials, colonies had not been treated with an acaricide for a minimum of 90 days before the trial began. In the nectar-dearth trial, the most recent treatment was oxalic acid vaporization. In the nectar-flow trial, the most recent treatment had been Apivar^®^.

Each trial was completed using thirty-six European-derived honey bee colonies of various strengths. We used twelve double deep colonies (at least twelve frames of brood and bees covering twenty frames), twelve single deep colonies (7–8 frames of brood and bees covering all 10 frames), and twelve nucleus colonies (4 frames of brood and bees covering all 5 frames). To achieve these uniform colony sizes, frames of bees and brood were moved between colonies during a two-week period before each trial began. Nucleus colonies were still housed in 10-frame equipment and allowed to grow without restraint during the trials. Each colony’s *V. destructor* populations were monitored via single alcohol washes every 3–4 weeks following standard procedures (33) for three months. Simply, a single alcohol wash was taken prior to treatment application as a baseline and then subsequently sampling occurred about once per month.

For the nectar-dearth trial, 18 of the 36 colonies (six double deep, six single deep, six nucleus) received the 4g active ingredient (ai)/L pouches, this concentration being predetermined by GreenLight Biosciences through separate research. Each pouch contained 500mL of the formulated product. The pouches are made of a foil-lined plastic bag material. Liquid is accessed by the bees from the top side of the pouch when laid flat on the top bars of the frames. The product label indicates pouches can also be placed on bottom boards, but we used the top bar application. Small, perforated holes in the pouch (which are covered with an adhesive sticker during transport and storage) allow the bees to use their proboscis to remove the product and transfer it to the brood chamber stores. Upon application, the double deep and single deep colonies received two pouches, and the nucleus colonies received one pouch. The other half of all the colonies served as negative controls, receiving pouches of a formulation blank (sucrose-based solution) that did not contain the active ingredient. A spacer rim (2.0 cm) was used to accommodate the extra height required. In each trial, we returned to the colonies three days after application to remove the empty pouches. Pouches were always completely emptied by the bees at this time, with no remaining product. At the time of treatment application, all colonies *V. destructor* infestations were measured via alcohol washes and the monitoring of mite populations continued every four weeks for three additional months. It should be noted that the dose applied (2 pouches per hive) to the double deep colonies is below the current approved directions for use, although this was not known at the time.

During this field trial, samples of *V. destructor* were taken from beneath fourth instar honey bee larvae. These mites targeted for sampling were submerged in brood food and hidden beneath the honey bee larvae. We expected that these mites were exposed to the dsRNA from the applied product at this stage. Brood food is a mixed diet of honey bee secretions and nectar/honey from the hive, and during the feeding of 4^th^-5^th^ instar larvae, the sugar content is approximately 47% (34). The nurse bees will use the applied sucrose-based solution as a component of this brood food, in turn exposing the mites to the dsRNA. Mites from beneath 4^th^ instar larvae were sampled on days 0 (immediately pre-application), 3, 7, and 14. Each hive was inspected to collect these mites; however, the number of mites obtained per hive varied from 1 to 10 based on infestation rate. These mites were immediately placed dry in 1.5mL tubes and stored on dry ice in the field until we returned to the lab and placed them in a -80 °C freezer the same day to assess gene knockdown in treated mites.

The nectar-flow trial used the same methods as the nectar-dearth trial. However, alcohol washes were collected to measure mite infestation every three weeks for three months post treatment. Also, a red dye (Sigma Aldrich; 458848-100G – 80%) was added to the sucrose-based solution of the treatments to trace its distribution and use in the hives.

#### 2.1.2 Dye tracing of Norroa™

**Figure 1:**
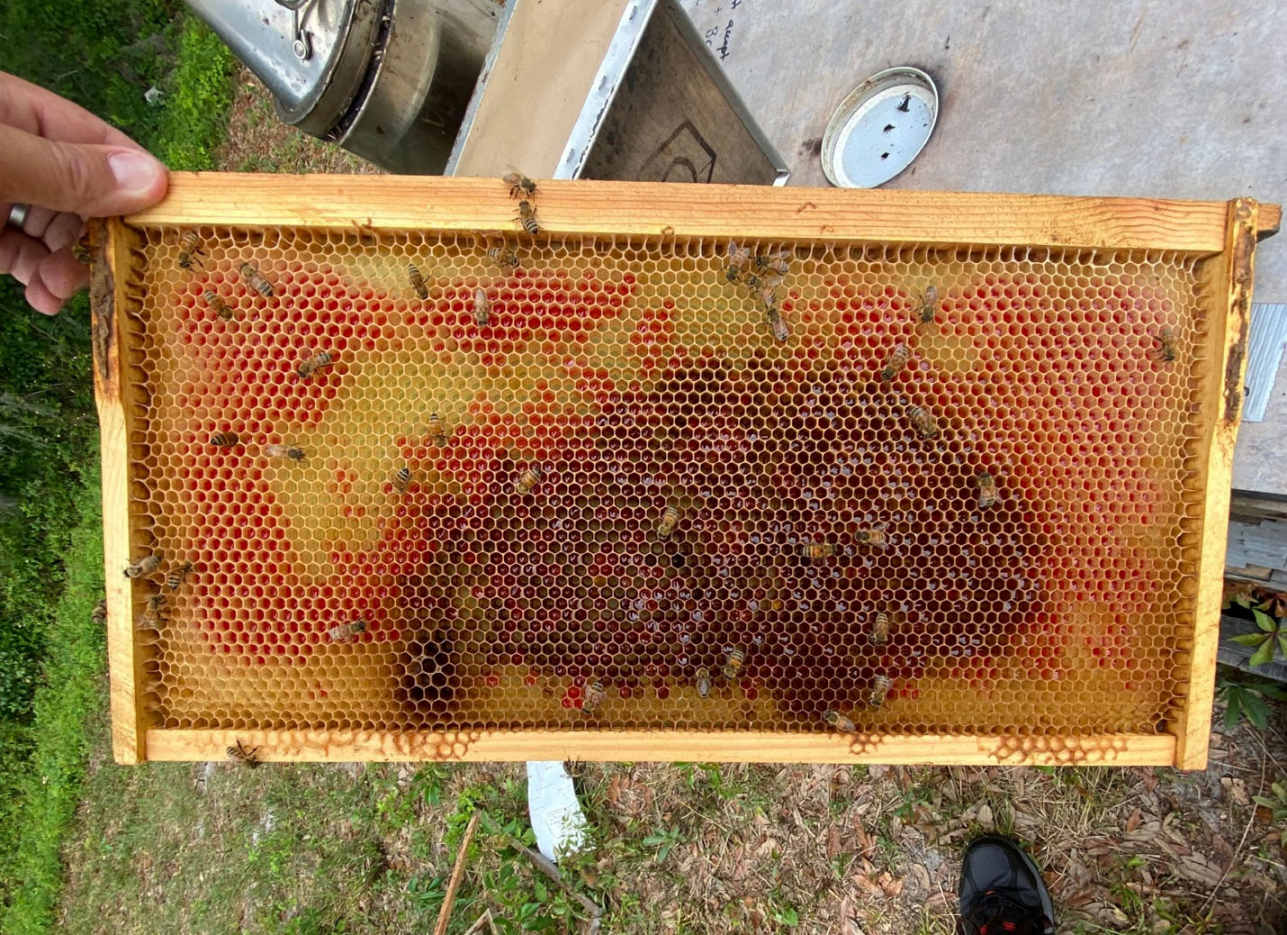
Photograph displaying the distribution of the dyed sugar syrup applied to experimental colonies that was stored in comb by worker honey bees.

During the nectar-flow trial, the product applied to each colony was colored with red dye (Allura red, 5% w/w) at a concentration of 4 mL/L in the pouches fed to the honey bee colonies, regardless of treatment (Details in **Supplementary Materials**). A spacer rim (2.0 cm) was used to accommodate the extra height required. During the process of honey bees taking and processing the contents of the pouch, the red dye could be used to trace its use in the hive (**Figure 1**). Samples of brood food were taken from all colonies at four distinct time points: pre-application (day 0), day 3, day 7, and day 14. These samples were stored at -80 °C until they could be analyzed.

##### 2.1.2.1 Concentrations of dye found in brood food

Allura red dye concentration in brood food was determined using a method described by Bişgin et al. (35) and Karatepe et al. (36), with some modifications. Briefly, approximately 19.5-20.5 mg of brood food was weighed into a 2 mL microcentrifuge tube, followed by the addition of 100 μL of deionized water. The mixture was briefly spun and vortexed for 10 seconds to homogenize. From this mixture, 50 µL of the diluted brood food was transferred into a new 2 mL microcentrifuge tube, and for each sample, 30 µL of 2 mol L^-^¹ NaCl, 160 µL of 6.25% Triton X-114, 100 µL of 2 mol L^-^¹ H_2_SO_4_, and 660 µL of distilled water were added sequentially with brief vortexing after each addition. The prepared samples were then incubated in a heat block at 60 °C for 30 min to induce phase separation. Following phase separation, the samples were immediately centrifuged (Sorvall Legend Micro 21R centrifuge, Thermo Fisher Scientific, Waltham MA) at 10,000 rpm for 5 minutes. The centrifuged tubes were placed on ice for 5 minutes to solidify the surfactant-rich phase. The upper dilute phase was decanted, and 1 mL of ethanol was added to the surfactant rich phase. The solution was vortexed for 10 seconds and after that filtered through a 0.45 µm PTFE syringe filter. 200 µL aliquots were transferred into wells of a 96-well microplate in duplicate and the absorbance was recorded at 506 nm using a Bio-Rad Benchmark plus microplate spectrophotometer (Bio-Rad, Hercules, CA). 1 mg/mL Allura red (5% w/w) stock solution was used to prepare working standards. Dye concentration in brood food extracts were calculated from the standard curve and expressed as µg dye per mg brood food.

#### 2.1.3 RNA extractions to determine the amount of target gene knockdown

**Figure 2:**
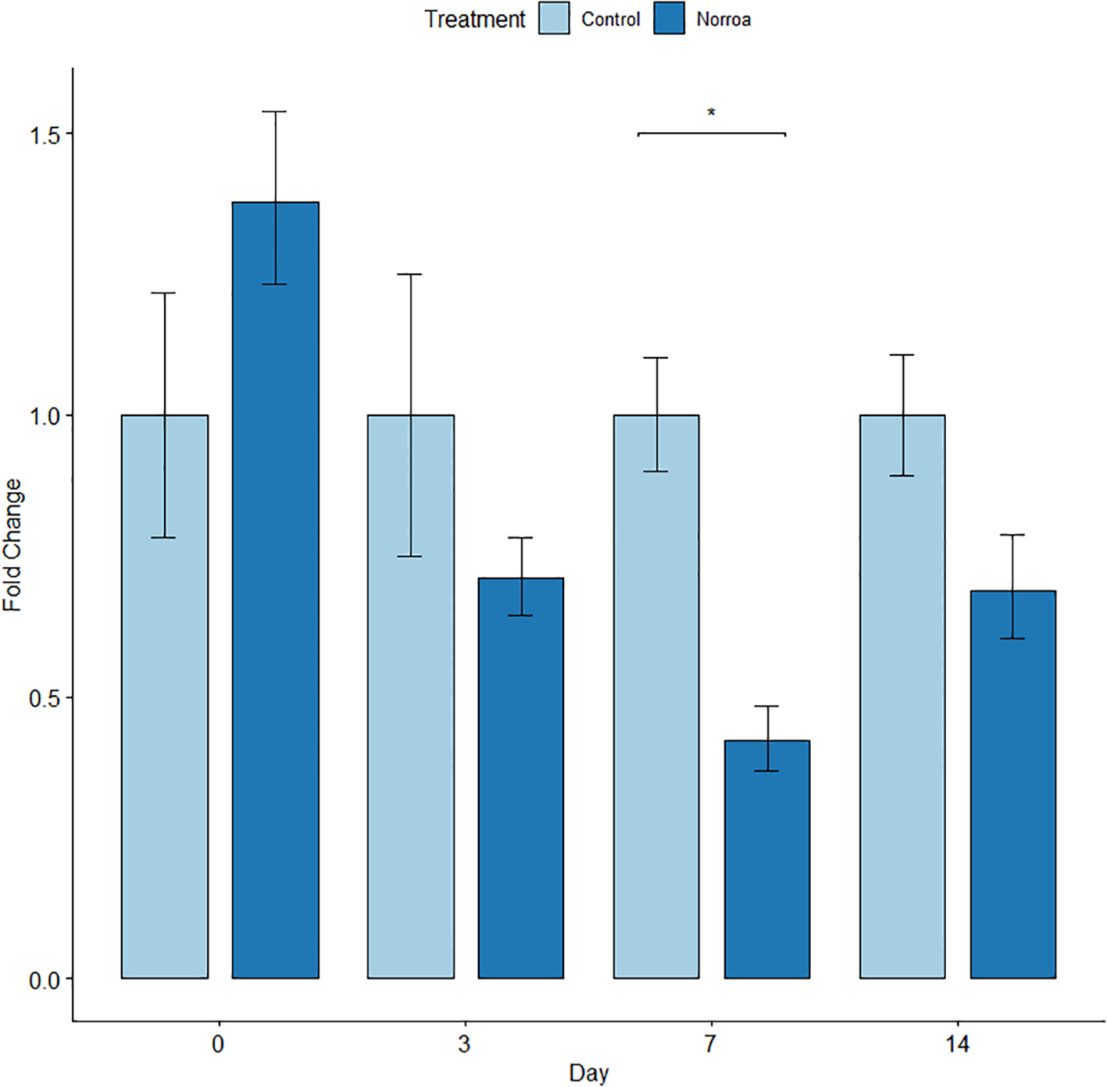
Fold change of *V. destructor* CAM expression Norroa™ treated and untreated honey bee colonies in the nectar-dearth field trial during Experiment 1. Bars are an average gene expression level of all mites combined from control colonies vs Norroa™ treated colonies. Sample sizes: Control day 0, n=4; day 3, n=10; day 7, n=16; day 14, n=7. Norroa™ day 0, n=10; day 3, n=10; day 7, n=24; day14, n=8. Asterisk represents significant differences. Bars represent means and error bars represent standard error.

Mites were homogenized using a GenoGrinder (SPEX SamplePrep, Model #2010115) and three 2.3 mm metal beads with cryo-blocks kept at -80 °C and run at a setting of 1350 for one minute. The number of mites collected and analyzed per hive ranged from one to six. Total RNA was extracted from homogenized samples using 500 µL of TRI-Reagent (Zymo, Cat. No. R2050-1-200) with the standard protocol. Bars in **Figure 2**, **3** represent an average expression level in all mites combined from control colonies sampled, normalized to 1. Then mites combined from sampled Norroa™ treated colonies relative gene expression levels.

**Figure 3:**
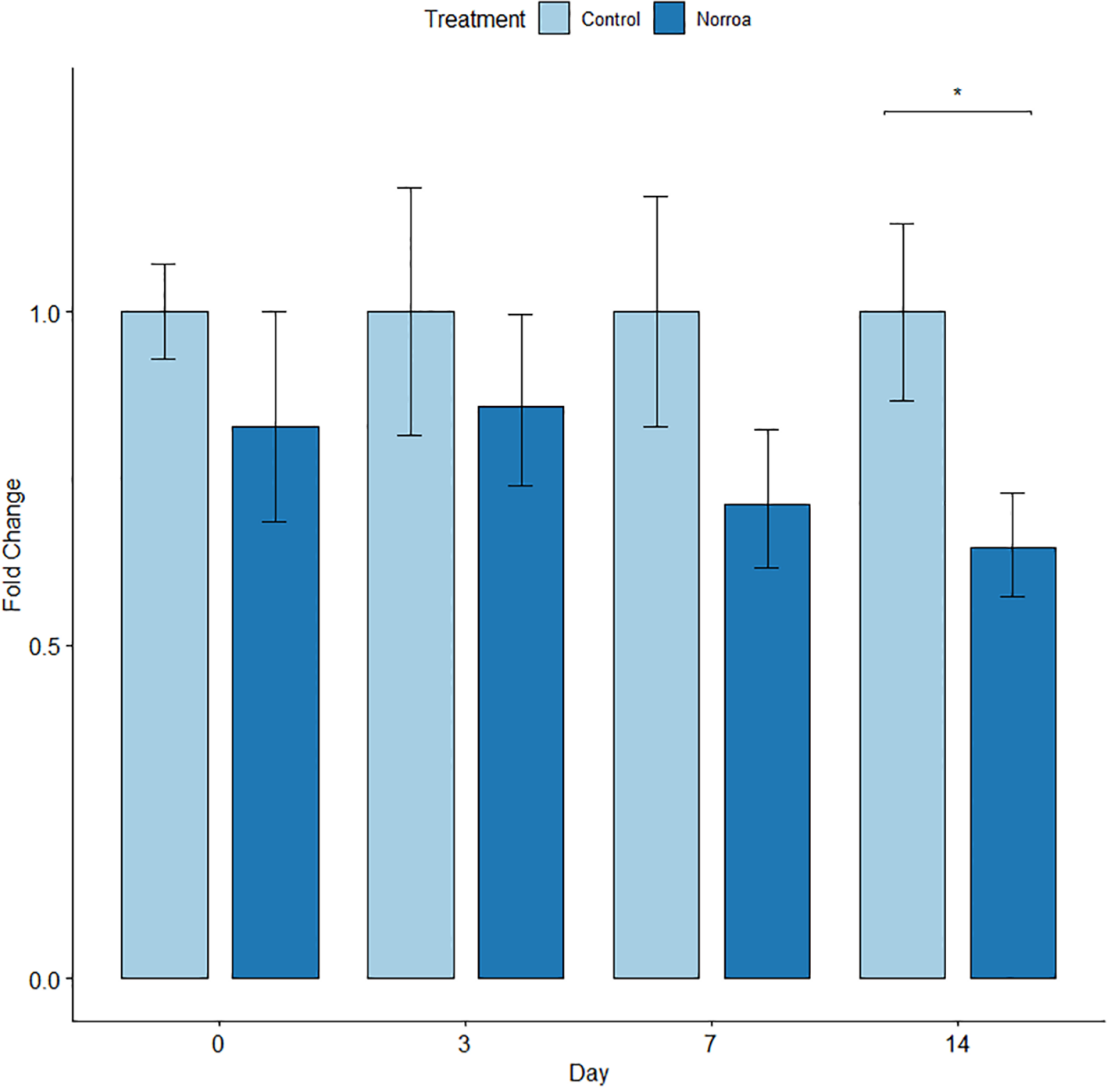
Fold change of *V. destructor* CAM expression Norroa™ treated and untreated honey bee colonies in the nectar-flow field trial during Experiment 1. Bars are an average gene expression level of all mites combined from sampling. Sample sizes: Control day 0, n=5; day 3, n=25; day 7, n=19; day 14, n=33. Norroa™ day 0, n=9; day 3, n=22; day 7, n=18; day 14, n=18. Asterisk represents significant differences. Bars represent means and error bars represent standard error.

#### 2.1.4 RT-qPCR

Total RNA concentrations were determined by NanoDrop 8000. RNA samples were normalized to a uniform concentration, and equal amounts were used for cDNA synthesis. M-MuLV Reverse Transcriptase (NEB, Cat. No. M0253L) was used in combination with Oligo -d(T)_20_VN (IDT, Cat. No. 51-01-15-08) for cDNA synthesis. qPCR was performed using Maxima SYBR Green 2x Master Mix (ThermoFisher, Cat. No. K0221) and using the standard protocol. Quantitative PCR was performed using a QuantStudio5 (Applied Biosystems) thermal cycler as follows: 95°C for 10 min, followed by 40 cycles of 95 °C for 15 sec and 60°C for one minute. Reactions were set in triplicate in 12 µL volumes.

Primers were designed specifically to detect the calmodulin-like (CAM-like) version of the calmodulin gene in *Varroa destructor* and tested to show an efficiency of 95.2%. The18S rRNA gene was used as the reference gene (37; See **Supplementary Table 1**).

### 2.2 Experiment 2

#### 2.2.1 *V. destructor* reproduction trial

In Experiment 2, we desired to observe the effect of Norroa™ to *V. destructor* reproduction within capped pupae cells. To make this observation, colonies were treated with either Norroa™ 4g/L or a formulation blank containing no active dsRNA. The treatment was applied as a pouch of sucrose-based solution and fed to the colonies directly on the top bar. Each pouch contained 500 mL, and two pouches were fed simultaneously to all our trial colonies. A spacer rim (2.0 cm) was used to accommodate the extra height required. The experiment began in October 2024 with 20 colonies – 10 were treated with Norroa™ and 10 were given a formulation blank control. Standard 10-frame Langstroth deep equipment were used, with all test colonies housed in a single deep box and having a population of at least eight frames of bees. These colonies were managed in the field before and throughout this experiment and had not received any acaricide treatment for three months prior to Norroa™ application.

We uncapped ~200–500 pupae in each colony, depending on mite infestation levels, 18 days after the initial application of Norroa™ (9–11 days post-capping of their cell) to examine reproductive success of those mites. Honey bee pupae with dark purple/black eyes and bodies were chosen to examine to ensure the greatest potential for the presence of several offspring of various stages. Any time a foundress mite was found, this was recorded. The inside of the cell and the entire pupa were examined to find any stages of *V. destructor* reproduction.

### 2.3 Statistical analysis

#### 2.3.1 Experiment 1 – Norroa™ field trials

All results were analyzed using R version 4.1.1. We initially analyzed the effects on nectar-dearth and nectar-flow trials using a linear mixed model (LMM) with a three-way interaction involving treatment (control vs. Norroa™ applied), application time, and colony size (nucleus, single, or double). Following the three-way interaction, we split colonies by size and tested the interaction between treatment and time for each of the three colony sizes. Similarly, at each time point, we tested the interaction between treatment and time. Analyses included colony as a random effect for all models. The difference between the final and initial times were calculated by subtracting the initial mite infestation rate from the final mite infestation rate. A two-way LMM with Gaussian error structures was used to compare the interactions of time and treatment on dye concentrations. The colony variable was used as a random effect for dye concentrations. In the absence of an interaction effect, multiple comparisons were conducted without the interaction effect in the model.

Reverse transcription-quantitative polymerase chain reaction (RT-qPCR) was used to measure gene knockdown fold change for CAM-like expression at different timepoints. Norroa™ treated samples were compared to controls for each day samples were taken. Fold changes were analyzed for both the nectar dearth and nectar flow experiments. Multiple comparisons were conducted for each time of sampling. RT-qPCR reactions produced threshold cycles (CT) for the CAM-like target gene and the 18S reference gene. The 2^-ΔΔ CT^ method was used to analyze relative expression of the CAM-like gene in Norroa™ treated samples.


ΔCT=CT(CAM−like gene)–CT(18S gene)

ΔΔCT=ΔCT(Norroa™ treatment)–ΔCT(syrup only treatment)

The *p*-value was produced by a two-tailed, type three Student’s T-test on the ΔCT values between the control group (Control) and the experimental group (Norroa™). Error bars are 95% confidence intervals.

#### 2.3.2 Experiment 2 – *V. destructor* reproduction trial

To test the proportion of cells that successfully reproduced, we used generalized linear mixed models (GLMMs) with binomial error structures. The model structures included treatment as the fixed effect and date sampled and colony source as random effects. All three criteria for reproduction were analyzed identically to each other.

## 3 Results

### 3.1 Experiment 1 - Norroa™ field trials

#### 3.1.1 Nectar-dearth

The average *V. destructor* infestation rate for all colonies at the beginning of this trial was at 4.56 mites/100 bees. Although *V. destructor* populations were numerically lower on average in treated colonies compared to the controls, we did not detect any significant differences between the treated and untreated colonies of any size (t = -1.02, p = 0.311). There were no significant effects of treatment, colony size or treatment × size on *V. destructor* populations when splitting the analysis by time (**Table 1**). When splitting the data by colony size, the only significant differences came from within the nucleus hives, where there were significantly higher mite populations at month three in the control group (**Table 1**). There were also significantly higher mite populations by the end of the experiment within the nucleus hive configuration compared to the beginning, with no significant effect of the treatments (**Table 1**). When evaluating the difference in *V. destructor* infestation rates from pre to post application, we still did not observe significant differences (**Table 1**).

**Table 1:** Main effects of the results in the nectar-dearth Norroa™ field trial during Experiment 1.

Response variable	Predictor variable	df	F-value	*P*-value
*Varroa destructor* population: time 1	Treatment	1	0.00	0.983
	Size	2	1.19	0.33
	Treatment × Size	2	0.17	0.918
*Varroa destructor* population: time 2	Treatment	1	0.55	0.463
	Size	2	0.41	0.745
	Treatment × Size	2	0.66	0.586
*Varroa destructor* population: time 3	Treatment	1	1.52	0.229
	Size	2	0.33	0.807
	Treatment × Size	2	0.69	0.565
Multi-comparison of deep hives	Treatment	1	0.55	0.468
	Time	2	0.7	0.512
	Treatment × Time	2	0.54	0.59
Multi-comparison of single hives	Treatment	1	0.21	0.651
	Time	2	2.33	0.116
	Treatment × Time	2	0.18	0.838
Multi-comparison of nucleus hives	Treatment	1	0.77	0.387
	**Time**	**2**	**13.81**	**<0.001**
	Treatment × Time	2	1.26	0.299
Difference between time 3 and time 1	Treatment	1	1.99	0.17
	Size	2	1.54	0.229
	Treatment × Size	2	0.63	0.601

All response variables were analyzed with treatment, time, and the interaction between the two as fixed effects and with the colony as a random effect. Significant results are in bold font.

#### 3.1.2 Nectar-flow trial

**Figure 4:**
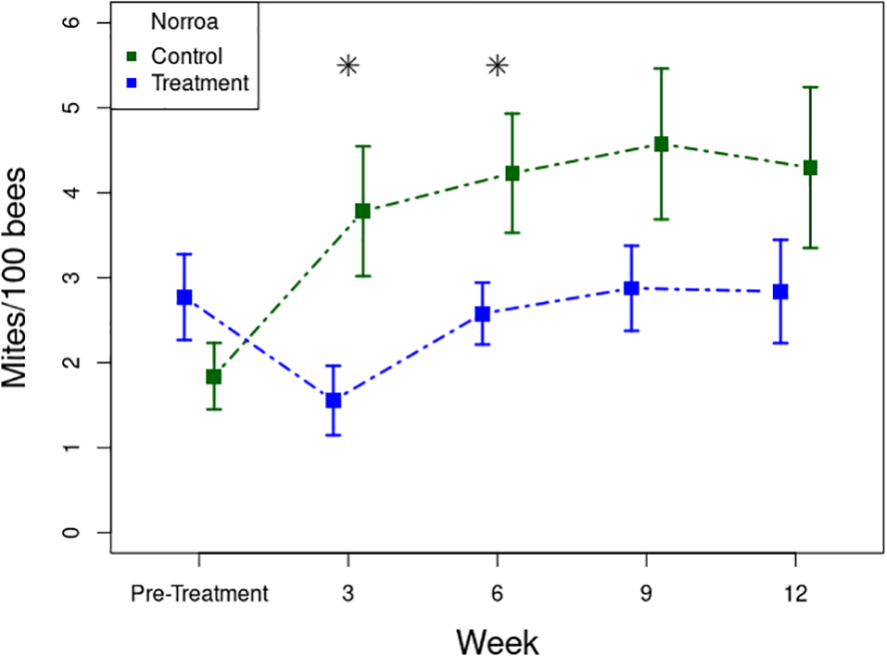
Mite infestation rates throughout the nectar-flow trial. Asterisks represent significant differences between treatment groups at a single time point.

The average *V. destructor* infestation rate for all colonies at the beginning of this trial was 2.31 mites/100 bees. On average, the colonies treated with Norroa™ maintained lower *V. destructor* infestations throughout the trial compared to the control treatment (F_1,147_ = 9.4, *p* = 0.003). Within hive configurations, the difference between treated and untreated colonies was only statistically significant in the double brood chamber colonies (F_1,47_ = 5.62, *p* = 0.022), which had significantly more mites per 100 bees than other sized groups (F_2,147_ = 6.56, *p* = 0.002). When comparing the difference from the final sampling point and the pre-treatment, the control group showed a significant increase in *V. destructor* infestation, and the treatment group remained unchanged (F_1,29_ = 6.37, *p* = 0.017) (**Figure 4**). Although there was no significant interaction between treatment and time (F_1,165_ = 2.69, *p* = 0.103), sampling weeks 3 and 6 post-treatment did show significant differences (Week 3: F_1,34_ = 6.61, *p* = 0.015; Week 6: F_1,33_ = 4.51, *p* = 0.041) (**Figure 4**).

#### 3.1.3 Concentrations of dye found in brood food

**Figure 5:**
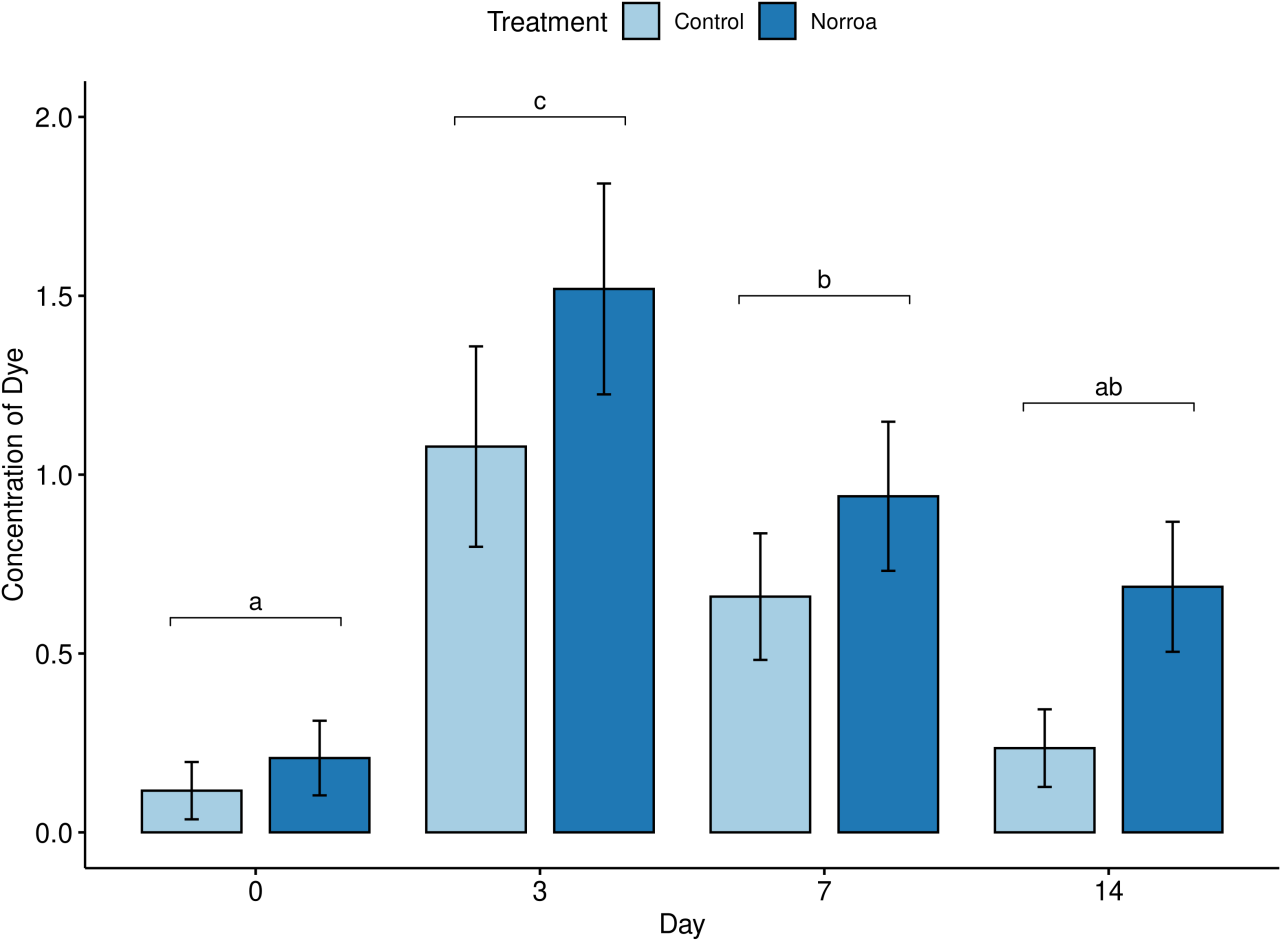
Mean concentration of dye (Allura red) in brood food over time in control and Norroa™. Bars with different lowercase letters indicate statistically significant differences using Tukey’s. All colonies fed dyed pouches. Control n = 18, Norroa™ n = 18. honest difference test at α = 0.05 for the day variable. Means are presented as (mean ± standard error).

No significant interaction was found in dye concentration over time between the treatment groups for brood food (F_3_,_119_= 0.4, *p* = 0.752). As all groups were fed dye, there were no significant differences in dye concentration observed between the treatment groups (F_1_,_119_ = 3.36, *p* = 0.075). However, there were significant differences in dye concentration observed over time (F_3_,_119_ = 15.51, *p*< 0.001; **Figure 5**). The concentration trended higher in brood food treated with Norroa™ compared to control. Over the sampling days, dye concentration was highest on Day 3 and gradually declined on day 7, reaching the lowest value on Day 14.

#### 3.1.4 Gene expression

##### 3.1.4.1 Nectar-dearth trial

There were significantly lower levels of CAM expression from the Norroa™ treatment relative to the control (58.1% lower) on day 7 (*p* = 0.0002) (**Figure 2**). While differences were not significantly different between treatments at days 3 and 14, CAM expression from the Norroa™ treatment relative to the control were reduced by 27% (*p* = 0.22) and 33% *p* = 0.057), respectively.

##### 3.1.4.2 Nectar-flow trial

There were significantly lower levels of CAM expression of the Norroa™ treatment compared to the control on day 14 (35.4% lower, *p* = 0.011) (**Figure 3**). Double (44.8%, *p* = 0.014) and single (51.1%, *p* = 0.016) deep sized colonies showed a significant reduction of CAM expression compared to the control. No other day showed significant differences between treatments (**Figure 3**).

### 3.2 Experiment 2 - *Varroa destructor* reproduction trial

Among tested colonies, 7707 cells were uncapped to find 320 mite infested cells. Treated colonies had 175 infested cells from 4806 uncapped. Control colonies had 145 infested cells from 2,901 uncapped. The average infestation rate of the control colonies was 5.0% and the average infestation rate of the treated colonies was 3.6%. These infestation rates are not an effect of the treatment, as the total population of mites would not have been affected by this time. Early in the experiment, two of the control colonies became queenless, and had minimal or inappropriate aged brood when it was time to uncap cells. Thus, we investigated ten treated colonies and eight control colonies.

There were significant differences in *V. destructor* reproduction between the Norroa™ treated and control colonies (**Table 2**). We observed 90.3% of *V. destructor* infested cells contained at least one egg or some kind of offspring in control colonies, but only 73.7% in the Norroa™ treated colonies (χ^2^ = 7.32, *p* = 0.007). If we limit the definition of successful reproduction to require a cell to have at least a nymph stage offspring (male or female), 82.8% of *V. destructor* infested cells contained reproducing mites in control colonies. However, the Norroa™ treated colonies had only 57.1% (χ^2^ = 9.37, *p* = 0.002). When comparing cells that had at least one male and one or more female nymph, we observed the Norroa™ treated colonies with 36% and the control group drop only slightly to 79.3% (**Table 2**; χ^2^ = 24.82, *p*< 0.001).

**Table 2:** Percent of cells containing a *V. destructor* foundress mite that had reproduced under three different criteria during Experiment 2. Values represent mean +/- standard error.

	Criteria for successful reproduction		
	At least 1 egg or other offspring	At least 1 nymph or other offspring	At least 1 male and 1 female nymph or other offspring
Norroa™ (n = 175)	73.7 ± 3.33	57.1 ± 3.75	36 ± 3.64
Control (n = 145)	90.3 ± 2.46	82.8 ± 3.15	79.3 ± 3.38
*P*-value	0.007	0.002	< 0.001

## 4 Discussion

Herein is the first published study in which the new *V. destructor* RNAi treatment Norroa™ has been tested in the United States. Our experiments demonstrated that there are promising aspects to using RNAi as a method of controlling *V. destructor* under various conditions, with some caveats in this study. For instance, we found that mites that were sampled from brood food after application of Norroa™ in Experiment 1 showed significantly lower gene expression, which would indicate effectiveness of the product in the short term. Furthermore, upon inspecting *V. destructor* infested cells in Experiment 2, we found significant reductions in viable offspring. Yet, during both the nectar-dearth and nectar-flow trials in Experiment 1, we did not observe uniform significant reductions of offspring across all treatments. Therefore, identifying the causes for this lower-than-expected efficacy should be considered and investigated further. One possible explanation could be that the starting mite numbers in both experiments were close to or above the normal treatment threshold (3 mites/100 bees (33). Given that the mode of action of vadescana reduces fecundity of the treated mites rather than killing adult (foundress) mites, it is more effective at maintaining low mite populations than reducing high mite populations. The results of these studies support this conclusion as mite populations did not increase in either field trial following treatment with Norroa™. In the nectar flow study (in which starting mite numbers were lower), there was a significant reduction in mite infestation relative to the untreated control.

In both the nectar-dearth and nectar flow trials in Experiment 1, we observed significant gene downregulation in recovered mites. Therefore, the active ingredient vadescana is having an impact on mites and suppressing calmodulin levels, as has been observed in laboratory assays (24). The level of reduction in gene expression demonstrates that an effective dose of dsRNA is reaching the mites.

Garbian et al. (23) investigated whether *V. destructor* populations could be reduced in mini hives (~600 bees) treated with dsRNA targeting genes different from vadescana. They found that they could reduce the mite populations by an average of about 60% after collecting all bees and mites remaining in the mini-hives. Recently, Bortolin et al. (26) conducted field tests with a mixture of several different dsRNAs to full sized colonies housed in single deep Langstroth hives and reduced *V. destructor* populations by about 40%. While we are not able to compare our findings directly to these studies, as we included larger honey bee colonies and more robust sampling techniques, we did observe significant decreases in *V. destructor* populations during our nectar-flow trial and reduced *V. destructor* reproduction in the field.

The current method of delivering the dsRNA product to the honey bee colonies appears to be effectively spreading into brood food throughout the hives as seen through our dyed treatment in Experiment 1. Nurse bees are characterized by well-developed hypopharyngeal glands (HPGs), also known as brood food glands located in the head. These glands are responsible for secretion of protein royal jelly that is essential for colony development. They serve as the primary food for larvae, queen, the drones and the workers (38, 39). In several tests, low-dose pesticide exposure did not change nursing behavior nor hypopharyngeal gland morphology and structural resilience in nurse bee hypopharyngeal glands (40–42). Likewise, treatment with Norroa™ does not appear to disturb the hypopharyngeal gland function. In our experiment, we observed a non-significant difference in Allura red concentrations between the dsRNA acaricide-treated and control colonies, suggesting that Norroa™ treatment does not affect nurse bee feeding or hypopharyngeal gland activity or any minor effects that may have been buffered at the colony level.

We did observe changes in dye concentration over time. On day 0, all brood samples were collected prior to dye exposure. However, a few samples showed positive values above detection point, reflecting background noise in the translucent samples rather than true dye presence. Further, some samples from day 3, day 7 and day 14 also fell below detection limit indicating that the material is not being distributed evenly to every cell. Among the detectable values, concentrations were observed to be higher on day 3 and then this level declined over time with lowest on day 14. This pattern overall suggests that the vadescana incorporation into brood food occurs shortly after delivery to the colony and later decreases over time as reserves in food stores are depleted. In our case dye was supplied through sugar syrup. Previous research, mostly from caged studies, has reported that sugar syrup consumption by honey bees increases initially and declines over time (43, 44). This feeding pattern may explain why we observe more dye level on Day 3 followed by decline. This decrease could be due to dilution or utilization of the food within the colony over time. Nurse bees may have consumed more supplemented food immediately after treatment resulting in higher dye incorporation into brood food. Although the protocol was optimized to enable dye detection in brood food, our results indicate that further refinement is still required to improve sensitivity and ensure consistent recovery across time.

We observed that the Norroa™ treatment was more effective at maintaining lower *V. destructor* infestation during the nectar-flow trial in Experiment 1 than during the nectar-dearth trial. In fact, at the end of the nectar-flow trial the Norroa™ group had *V. destructor* rates below 3 mites per 100 bees after 12 weeks of monitoring, compared to the control colonies which grew to 4.5 mites per 100 bees after only 9 weeks. The most notable difference between the two trials was the initial *V. destructor* infestation rates, with the nectar-dearth trial beginning with nearly twice the infestation rate as the nectar-flow trial. Due to this high starting *V. destructor* population during the nectar-dearth trial, the only significant change was within the nucleus hive configuration, where their mite populations were extremely high by the end of the experiment. It is likely because of the high *V. destructor* infestation during the nectar-dearth trial at the time of application, mites were reproducing at such a rate that a reduction in infestation was not possible. In Florida where our experiment was conducted, *V. destructor* populations naturally grow incredibly rapidly during the summer and autumn seasons, when honey bee brood is being produced by the honey bee colonies (4). Conversely, during the spring season when the nectar-flow trial was conducted, we would expect a slower *V. destructor* population growth (4). Therefore, with a lower starting infestation rate during the season when *V. destructor* populations do not grow as quickly, the mite population growth could be effectively reduced by the treatment. Most chemical-based *V. destructor* treatments are least effective during the Florida summer and autumn seasons (45). As a result of the mode of action of vadescana resulting in reduced fecundity of reproductive foundress mites rather than killing adult phoretic mites (24), Norroa™ is more effective at preventing the exponential growth of mite infestations than it is at reducing high mite infestations. Thus, this the current formulation of the Norroa™ treatment could best be used in the spring or when mite populations are below a damaging threshold.

Our Experiment 2 field trial investigating mite reproduction in cells showed the clearest results of the RNAi effect on mite populations. There was significant inhibition of advanced mite reproduction amongst the treated colonies. Interestingly, when the cells containing pupae were observed, 9–11 days post-capping, there was a much higher proportion of cells that contained mite eggs in the treated colonies. This is important to note because at this late stage of honey bee pupae development, we do not expect to see mite eggs which are typically laid in the first six days after capping (46, 47). It is possible that these eggs were laid during the expected period but were not viable, thus remaining in their egg state until our observations. Alternatively, it is possible that the egg laying of the foundress mite was delayed such that we were still able to observe eggs so late in the honey bee pupae development. If we consider that the offspring needed for the mite to have effectively reproduced is to have both a male and a female mite in at least a nymph stage, then the Norroa™ treatment reduced mite reproduction by >40% in Experiment 2 (**Table 2**).

Our results demonstrate that Norroa™ is being distributed in the honey bee colony and is being incorporated into brood food based on the dye study. The results presented here further indicate that the target gene is being down-regulated in treated mites, and that fecundity of treated mites is being reduced in full sized colonies treated in the field. The level of reduction of mite reproduction reported here (>40%) is lower than in previously reported laboratory studies (24), but this is likely due to dilution with other nectar sources and uneven distribution to the cells, as there are other nectar sources in the field compared to Norroa™ being the sole nectar source in a laboratory study.

All around the world beekeepers are desperate for effective *V. destructor* controls. Although research related to biological methods are currently under-studied relative to chemical treatments (32), researchers continue to discover novel RNA targets of key *V. destructor* genes that can be affected by RNAi (28, 48, 49). Thus, future research efforts should focus on optimizing this Norroa™ product while field testing new dsRNA active ingredients, so there are more RNAi treatment options available to beekeepers. It is essential that beekeepers utilize different chemical and non-chemical treatments for a sustainable approach to *V. destructor* control (7). Part of a sustainable IPM system is rotating chemical treatments with products that have varying modes of action. Using an RNAi-based product like Norroa™ provides a new safe and effective mode of action for beekeepers.

## Data Availability

The raw data supporting the conclusions of this article will be made available by the authors, without undue reservation.
