# Supplementary material for: Evaluating the new product Norroa™ against Varroa destructor in managed honey bee (Apis mellifera) colonies
Source: Front Insect Sci. 2026 Mar 11;6:1751606. doi: 10.3389/finsc.2026.1751606 (PMC13013518; doi:10.3389/finsc.2026.1751606)
Supplement: Supplementary file 1 [file Table1.docx]

Primers for RT-qPCR:

| **Target Gene** | **For Primer** | **Forward Sequence** | **Rev Primer** | **Reverse Sequence** |
| --- | --- | --- | --- | --- |
| 18S rRNA (18S) | Vd_18S_F | AATGCCATCATTACCATCCT | Vd_18S_R | CAAAAACCAATCGGCAATCT |
| CAM-like | Vd_CAM_F2 | CAGCACTACCTGATACAGCGAC | Vd_CAM_R2 | GCAGTTTGCACAGTCCGTTC |
